# Structural and functional diversity calls for a new classification of ABC transporters

**DOI:** 10.1002/1873-3468.13935

**Published:** 2020-10-26

**Authors:** Christoph Thomas, Stephen G. Aller, Konstantinos Beis, Elisabeth P. Carpenter, Geoffrey Chang, Lei Chen, Elie Dassa, Michael Dean, Franck Duong Van Hoa, Damian Ekiert, Robert Ford, Rachelle Gaudet, Xin Gong, I. Barry Holland, Yihua Huang, Daniel K. Kahne, Hiroaki Kato, Vassilis Koronakis, Christopher M. Koth, Youngsook Lee, Oded Lewinson, Roland Lill, Enrico Martinoia, Satoshi Murakami, Heather W. Pinkett, Bert Poolman, Daniel Rosenbaum, Balazs Sarkadi, Lutz Schmitt, Erwin Schneider, Yigong Shi, Show-Ling Shyng, Dirk J. Slotboom, Emad Tajkhorshid, D. Peter Tieleman, Kazumitsu Ueda, András Váradi, Po-Chao Wen, Nieng Yan, Peng Zhang, Hongjin Zheng, Jochen Zimmer, Robert Tampé

**Affiliations:** 1 Institute of Biochemistry, Biocenter, Goethe University Frankfurt, Germany; 2 Department of Pharmacology and Toxicology, University of Alabama at Birmingham, AL, USA; 3 Department of Life Sciences, Imperial College London, London South Kensington, UK; 4 Rutherford Appleton Laboratory, Research Complex at Harwell, Didcot, UK; 5 Structural Genomics Consortium, University of Oxford, UK; 6 Skaggs School of Pharmacy and Pharmaceutical Sciences and Department of Pharmacology, School of Medicine, University of California, San Diego, La Jolla, CA, USA; 7 State Key Laboratory of Membrane Biology, Institute of Molecular Medicine, Beijing Key Laboratory of Cardiometabolic Molecular Medicine, Peking University, Beijing, China; 8 Peking-Tsinghua Center for Life Sciences, Peking University, Beijing, China; 9 Institut Pasteur, Paris Cedex 15, France; 10 Division of Cancer Epidemiology and Genetics, National Cancer Institute, NIH, Gaithersburg, MD, USA; 11 Department of Biochemistry and Molecular Biology, Faculty of Medicine, Life Sciences Institute, University of British Columbia, Vancouver, BC, Canada; 12 Department of Cell Biology and Department of Microbiology, New York University School of Medicine, NY, USA; 13 Faculty of Biology, Medicine and Health, The University of Manchester, UK; 14 Department of Molecular and Cellular Biology, Harvard University, Cambridge, MA, USA; 15 Department of Biology, Southern University of Science and Technology, Shenzhen, China; 16 Institute for Integrative Biology of the Cell (I2BC), Université Paris-Sud, Orsay, France; 17 National Laboratory of Biomacromolecules, CAS Center for Excellence in Biomacromolecules, Institute of Biophysics, Chinese Academy of Sciences, Beijing, China; 18 Department of Chemistry and Chemical Biology, Harvard University, Cambridge, MA, USA; 19 Institute for Integrated Cell-Material Sciences (WPI-iCeMS), Kyoto University, Japan; 20 Department of Pathology, University of Cambridge, UK; 21 Structural Biology, Genentech Inc., South San Francisco, CA, USA; 22 Division of Integrative Bioscience and Biotechnology, POSTECH, Pohang, Korea; 23 Department of Biochemistry, The Bruce and Ruth Rappaport Faculty of Medicine, The Technion-Israel Institute of Technology, Haifa, Israel; 24 Institut für Zytobiologie, Philipps-Universität Marburg, Germany; 25 Department of Plant and Microbial Biology, University Zurich, Switzerland; 26 International Research Centre for Environmental Membrane Biology, Foshan University, Foshan, China; 27 Department of Life Science, Tokyo Institute of Technology, Yokohama, Japan; 28 Department of Molecular Biosciences, Northwestern University, Evanston, IL, USA; 29 Department of Biochemistry, Groningen Biomolecular Sciences and Biotechnology Institute, University of Groningen, The Netherlands; 30 Department of Biophysics, University of Texas Southwestern Medical Center, Dallas, TX, USA; 31 Institute of Enzymology, Research Center for Natural Sciences (RCNS), Budapest, Hungary; 32 Institute of Biochemistry, Heinrich Heine University Düsseldorf, Düsseldorf, Germany; 33 Department of Biology/Microbial Physiology, Humboldt-University of Berlin, Germany; 34 Institute of Biology, Westlake Institute for Advanced Study, School of Life Sciences, Westlake University, Hangzhou, China; 35 Department of Chemical Physiology and Biochemistry, Oregon Health & Science University, Portland, OR, USA; 36 Department of Biochemistry, Center for Biophysics and Quantitative Biology, NIH Center for Macromolecular Modeling and Bioinformatics, Beckman Institute for Advanced Science and Technology, University of Illinois at Urbana-Champaign, IL, USA; 37 Department of Biological Sciences and Centre for Molecular Simulation, University of Calgary, AB, Canada; 38 Institute for Integrated Cell-Material Sciences (WPI-iCeMS), KUIAS, Kyoto University, Japan; 39 Department of Molecular Biology, Princeton University, NJ, USA; 40 National Key Laboratory of Plant Molecular Genetics, CAS Center for Excellence in Molecular Plant Sciences, Institute of Plant Physiology and Ecology, Shanghai Institutes for Biological Sciences, Chinese Academy of Sciences, Shanghai, China; 41 Department of Biochemistry and Molecular Genetics, School of Medicine, University of Colorado Anschutz Medical Campus, Aurora, CO, USA; 42 Molecular Physiology and Biological Physics, University of Virginia School of Medicine, Charlottesville, VA, USA

**Keywords:** ABC transporters, ATPases, cryo-EM, membrane proteins, molecular machines, phylogeny, primary active transporters, sequence alignment, structural biology, X-ray crystallography

## Abstract

Members of the ATP-binding cassette (ABC) transporter superfamily translocate a broad spectrum of chemically diverse substrates. While their eponymous ATP-binding cassette in the nucleotide-binding domains (NBDs) is highly conserved, their transmembrane domains (TMDs) forming the translocation pathway exhibit distinct folds and topologies, suggesting that during evolution the ancient motor domains were combined with different transmembrane mechanical systems to orchestrate a variety of cellular processes. In recent years, it has become increasingly evident that the distinct TMD folds are best suited to categorize the multitude of ABC transporters. We therefore propose a new ABC transporter classification that is based on structural homology in the TMDs.

We suggest a new classification of the ABC transporter superfamily that is based on the TMD fold. Historically, first hints of the ABC protein superfamily came from sequence alignments of bacterial proteins that revealed highly conserved motifs in their ATPase domains [1]. The superfamily of ABC proteins was subsequently divided into three main classes [2-4]: exporters, nontransporter ABC proteins, and a third class consisting primarily of importers. The mammalian ABC systems, in particular, were grouped into seven subfamilies (ABCA to ABCG), based on NBD and TMD sequence homology, gene structure, and domain order [5-7]. It should be noted that ABCE and ABCF are not transporters, but exist as twin-NBDs without TMDs and are involved in mRNA translation control [8]. Detailed membrane topology and sequence analyses of exporters uncovered that, in contrast to the NBDs, the TMDs are polyphyletic and can serve as references to categorize ABC transporters into three distinct types (ABC1-3) [9,10]. According to this classification, the cystic fibrosis transmembrane conductance regulator (CFTR), the transporter associated with antigen processing (TAP), and the drug efflux pump P-glycoprotein (P-gp) belong to the ABC1 transporters; ABCG2 and ABCG5/G8 are members of the ABC2 group, which also comprises importers; and the macrolide translocator MacB is categorized as an ABC3 system. Yet, another classification scheme currently in use differentiates between the three types of importers predominantly found in prokaryotes [11-14] and two types of exporters, exemplified by Sav1866 [15] and ABCG5/8 [16], in addition to the LptB_2_FG-type [17,18] and MacB-type [19-22] transporters.

Our motivation for proposing a revised nomenclature stems from the recent wealth of ABC transporter structures determined by X-ray crystallography and single-particle cryo-electron microscopy, which has unveiled a remarkable diversity of TMD folds and evolutionary relationships between bacterial and eukaryotic/mammalian transporters [16-21,23-26]. This affluence of structural information provides the opportunity to introduce a universal nomenclature that combines previous phylogenetic analyses with the new findings coming from high-resolution structures. The nomenclature groups ABC transporters into distinct types, I–VII, based on their TMD fold (Fig. 1, Tables 1 and 2). This classification is supported by quantitative analyses using TM-scores based on pairwise structural alignment of TMDs (Tables S1-S6, Fig. S1). The classification focuses on the transporter-forming TMDs and does not consider additional membrane integrated domains, as for example observed in TAP1/TAP2 [27,28].

As before, types I-III of the new nomenclature cover the three different importer architectures (Fig. 1, Table 1, Tables S2 and S3; TM-score for pairwise structural alignment between the type III systems CbiQ (PDB code 5X3X) and EcfT from *Lactobacillus brevis* (PDB code 4HUQ): 0.736). It is noteworthy that prokaryotic importers typically operate with periplasmic, extracellular, or membrane-embedded substrate-binding proteins whose structural features correlate with the type of TMD fold [29].

Based on the characteristic structure of the founding member Sav1866, which includes a domain-swapped TMD arrangement, type IV members of the new nomenclature have previously been classified as type I ABC exporters [15]. However, a significant and growing number of these ABC proteins have nonexporter functions, i.e., the gated chloride channel CFTR, the regulatory K_ATP_ channel modules SUR1/2, the lysosomal cobalamin (vitamin B_12_) transporter ABCD4 [30], the bacterial siderophore importers YbtPQ and IrtAB, and the cobalamin/antimicrobial peptide importer Rv1819c [31-33], as well as several type IV systems with importer functions in plants [34-39]. This striking functional diversity mediated by the same structural framework (Fig. 1, Tables 1 and 2, Tables S4 and S5) makes the type IV ABC transporters stand out and is also the main reason why we suggest the more universal taxonomy based on structural principles.

According to the new classification, type V systems are ABC transporters of the ABCG/ABCA/Wzm type (Fig. 1, Tables 1 and 2, Table S6). They include channel-forming biopolymer secretion systems in bacteria [25,26]. Remarkably, although many type V systems are exporters, this type also comprises transporters with import function, including the retina-specific importer (flippase) ABCA4 (rim protein) [40,41] and importers in plants [42-44].

Finally, LptB_2_FG and MacB are the founding members of type VI and type VII ABC transporters, respectively. We are aware that LptF and LptG have TMD folds that resemble type V members, and the TMD of MacB is reminiscent of type V systems and LptF/G. Yet, they exhibit distinct features that warrant classifications into separate groups. These include the lack of an amphipathic N-terminal ‘elbow helix’ and no extracellular reentrant helices between TM5 and TM6. In addition, MacB contains only four proper TM helices as well as an additional coupling helix, thereby defining a separate transporter architecture. In accordance with differences in TMD topologies, the LptFG and MacB transporters also display diverging dimerization interfaces. Thus, we have chosen to assign LptFG and MacB to separate types. This notion is corroborated by the TM-score-based quantitative analysis (Table S6 and Fig. S1). Of note, at the time of writing, publicly available, yet unpublished structures of the lipid transporter complex MlaFEDB of *Gram*-negative bacteria reveal some resemblance of MlaE to LptF/G and MacB. However, the number of TM helices differs between LptFG (six TM helices), MlaE (five TM helices), and MacB (four TM helices) [45-48] (Table S6 and Fig. S1).

We would like to point out that the classification of the mammalian ABC transporters into the ABCA-G subfamilies can be maintained as subcategories of type IV (subfamilies B–D) and type V (subfamilies A and G) within the new nomenclature (Table 2). We are also not proposing any changes to gene symbols. Most importantly, the new nomenclature based on TMD architecture can be universally applied to ABC transporters beyond their particular physiological functions and across the three domains of life. Hence, it allows any newly discovered transporter fold to be compared with the existing types and seamlessly incorporated into the classification scheme, possibly as a new type. Since the new nomenclature depends on TMD architecture, it requires structural information in order to classify new transporter systems. At the same time, we regard the nomenclature as a dynamic platform that can be upgraded, adjusted, or refined whenever necessary due to novel insights that add extra dimensions to our understanding of ABC systems.

The recent advances in structural mapping of the diverse superfamily of ABC transporters have revealed a vast area of mechanistically uncharted territory. One key objective of future research should be to fully comprehend how type IV systems perform so many different functions, i.e., as importer, exporter, lipid floppase, ion channel, and regulator, by employing a single structural scaffold. However, we do not exclude that other types might turn out to be as functionally diverse as type IV systems. Exploring the different modes of operation and accompanying conformational landscapes [49] and the dynamics of the multifarious ABC systems will require integrative experimental approaches that include electron paramagnetic resonance (EPR), nuclear magnetic resonance (NMR), single-molecule techniques, and single-turnover experiments. We are confident that future studies of such kind will provide major new insights into the mechanisms of these fascinating molecular machines.

## Supplementary Material

Fig. S1. Phylogenetic tree based on TM-scores of structural TMD alignments.

Supplementary Material **Table S1.** TM-scores based on pairwise structural alignment of representatives of the different TMD types.
**Table S2.** TM-scores based on pairwise structural alignment of type I TMDs.
**Table S3.** TM-scores based on pairwise structural alignment of type II TMDs.
**Table S4.** TM-scores based on pairwise structural alignment of type IV TMDs in inward-facing conformations.
**Table S5.** TM-scores based on pairwise structural alignment of type IV TMDs in (semi-) occluded/outward-facing conformations.
**Table S6.** TM-scores based on pairwise structural alignment of type V, VI, and VII TMDs^a^.

## Figures and Tables

**Fig. 1.**
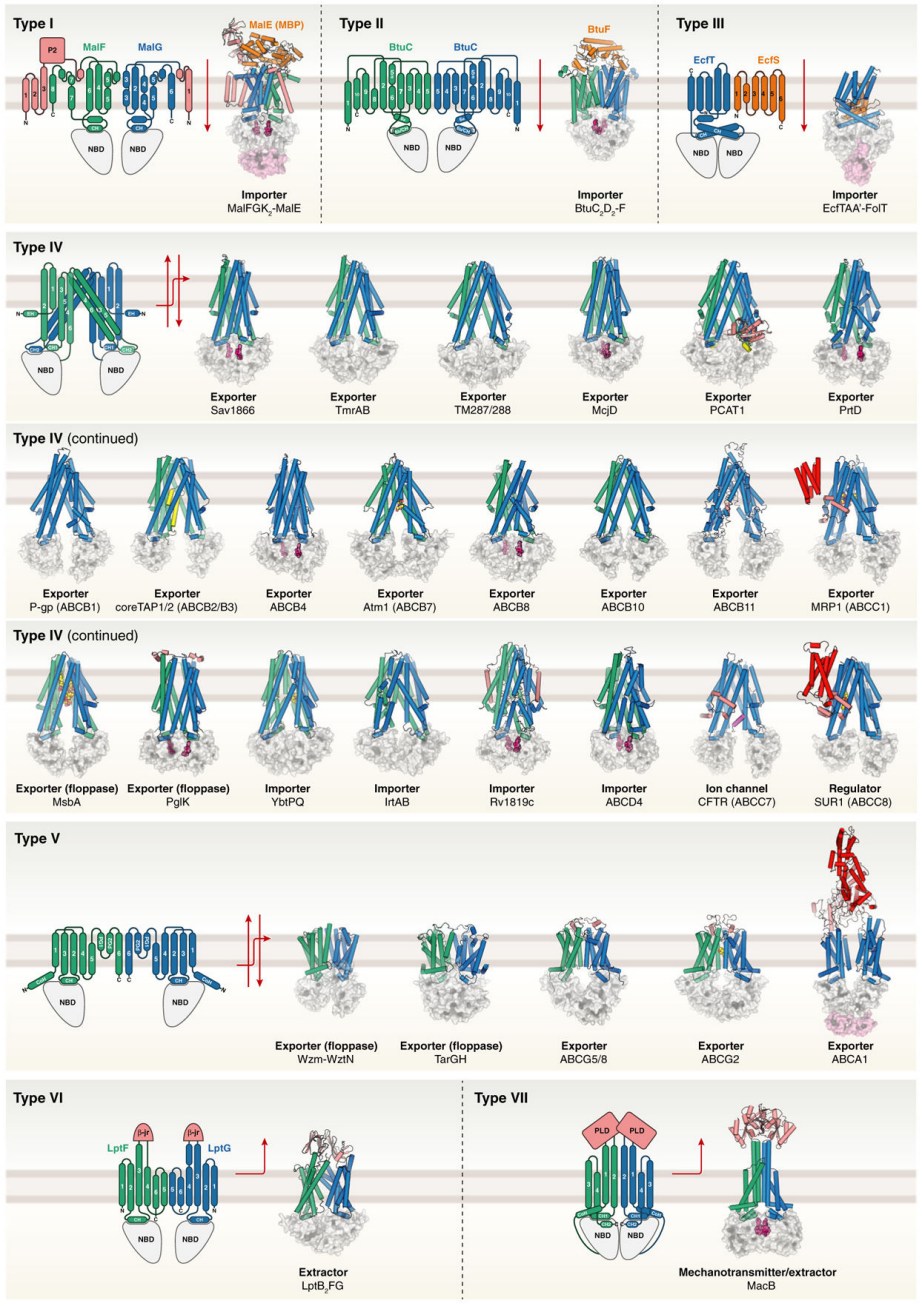
The different types within the ABC transporter superfamily. Members of the superfamily of ABC transporters can be grouped into distinct types based on their TMD fold. The TMDs of representative experimentally determined structures are depicted as cartoons, and their NBDs are shown in surface representation. The TMD architecture of the first structure of each type is illustrated by a topology diagram. The number of structures shown for each transporter type does not necessarily reflect the abundance or importance of the respective type, but highlights the common scaffold and functional diversity of the transporters. The two TMDs of each transporter are shown in green and blue, respectively, except for cases where the TMDs are part of the same polypeptide chain (uniform blue color). Please note that the type V ABC transporters also include the retina-specific importer ABCA4 and importers in plants. Substrate-binding components of type I-III folds are illustrated in orange, and auxiliary domains and additional (TM) helices are shown in red, salmon, and violet, respectively. Bound (occluded) nucleotides and Mg^2+^ ions in the NBDs are shown as dark pink spheres. Transported substrates and inhibitors are shown in yellow (carbon) and in CPK colors (remaining atoms in small-molecule compounds), respectively. The directions of substrate transport are indicated by solid and dashed red arrows. The structures have the following Protein Data Bank (PDB) accession codes: MalFGK_2_-MalE: 2R6G [12]; BtuC_2_D_2_-BtuF: 4FI3 [50]; EcfTAA′-FolT: 4HUQ [14]; Sav1866: 2HYD [15]; TmrAB: 5MKK [51]; TM287/288: 4Q4H [52]; McjD: 4PL0 [53]; PCAT1: 6V9Z [54]; Atm1: 4MYH [55]; MRP1: 5UJA [56]; PrtD: 5L22 [57]; P-gp: 4M1M [58]; TAP1/2: 5U1D [59]; ABCB4: 6S7P [60]; ABCB8: 5OCH; ABCB10: 3ZDQ [61]; ABCB11: 6LR0 [62]; MsbA: 5TV4 [63]; PglK: 6HRC [64]; YbtPQ: 6P6J [31]; IrtAB: 6TEJ [32]; Rv1819c: 6TQF [33]; ABCD4: 6JBJ [30]; CFTR: 5UAK [65]; SUR1: 6BAA [66]; Wzm-WztN: 6OIH [25]; TarGH: 6JBH [26]; ABCG5/8: 5DO7 [16]; ABCG2: 6HCO [67]; ABCA1: 5XJY [23]; LptB_2_FG: 5X5Y [17]; MacB: 5LJ7 [21]. ABC, ATP-binding cassette; β-jr, β-jellyroll-like domain; C, C terminus; CH, coupling helix; CoH, connecting helix; EH, elbow helix; N, N terminus; NBD, nucleotide-binding domain; P2, extracytoplasmic loop; PG, periplasmic gate helix; PLD, periplasmic domain; TMD, transmembrane domain.

**Table 1.** Prokaryotic ABC transporters classified according to their TMD folds.

TMD fold	TM helix organization	Experimentally determined structures	PDB codes^a^	Function
Type I	(**5**-6) + (**5**-6/8)^b^	MalFGK_2_(-E)	2R6G, 3FH6, 3PUV, 3PUW, 3PUX, 3RLF, 4JBW	Maltose import
		ModB_2_C_2_(-A)	2ONK, 3D31	Molybdate import
		MetNI(-Q)	3DHW, 3TUI, 3TUJ, 3TUZ, 6CVL	Methionine import
		Art(QN)_2_	4YMS, 4YMT, 4YMU, 4YMV, 4YMW	Amino acid import
		AlgM1M2SS-Q2	4TQU	Alginate import
Type II	10 + 10	BtuC_2_D_2_(-F)	1L7V, 2QI9, 4DBL, 4FI3, 4R9U	Cobalamin import
		MolBC	2NQ2	Import of molybdate and tungstate
		HmuUV	4G1U	Heme import
		BhuUV(-T)	5B57, 5B58	Heme import
Type III	4-8 (T) + 6-7 (S)	EcfTAA′-Fo1T	4HUQ, 5D3M, 5JSZ	Folate import
		EcfTAA′-PdxU2	4HZU	Pyridoxine import
		*Lb*ECF-PanT	4RFS	Pantothenate import
		CbiMQO	5X3X, 5X41	Co^2+^ import
		ECF-CbrT	6FNP	Cobalamin import
Type IV	6 + 6 Homodimer Heterodimer Single chain	Sav1866	2HYD, 2ONJ	Multidrug export
		MsbA	3B60, 3B5Y, 3B5Z, 5TV4, 6BPL, 6BPP, 6BL6, 6O30, 6UZ2, 6UZL	Lipid A/LPS flopping
		*Na*Atm1	4MRR, 4MRS, 4MRV, 4MRN, 4MRP	Export of GSH, GSH-related compounds, and metal-GSH complexes
		TM287/288	4Q4A, 4Q4H, 4Q4J, 6QUZ, 6QV0, 6QV1, 6QV2	Daunorubicin export
		McjD	4PL0, 5EG1, 5OFR	Antimicrobial peptide export
		PCAT1	4RY2, 6V9Z	Polypeptide export
		PglK	5C76, 5C78, 5NBD, 6HRC	Export (flopping) of lipid-linked oligosaccharides
		TmrAB	5MKK, 6RAF, 6RAG, 6RAH, 6RAI, 6RAJ, 6RAK, 6RAL, 6RAM, 6RAN	Peptide export
		PrtD	5L22	Polypeptide type-1 secretion system
		YbtPQ	6P6I, 6P6J	Metal–siderophore import
		Rv1819c	6TQE, 6TQF	Import of cobalamin and bleomycin
		IrtAB	6TEJ	Iron–siderophore import
Type V	6 + 6 Homodimer Heterodimer Single chain	Wzm-WztN TarGH	6OIH, 6M96	O-antigen export (flopping)
		TarGH	6JBH	Export (flopping) of wall teichoic acid
Type VI	6 + 6 Heterodimer	LptB_2_FG(C)	5X5Y, 5L75, 6MIT, 6MJP, 6MHU, 6MHZ, 6MI7, 6MI8, 6S8G, 6S8H, 6S8N	LPS extraction
Type VII	4 + 4	MacB	5GKO, 5WS4, 5LIL, 5LJ6, 5LJ7, 5XU1	Export of macrolides and polypeptide virulence factors

GSH, glutathione; LPS, lipopolysaccharide.

a Only PDB codes of structures with an overall resolution equal to or better than 4.5 Å were included.

b Conserved TMs in bold.

**Table 2.** Eukaryotic ABC transporters classified according to their TMD folds^a^.

TMD fold	TM helix organization	Experimentally determined structures	PDB codes^b^	Function
Type IV	6 + 6 Homodimer Heterodimer Single chain	ABCB subfamily		
		P-gp (ABCB1)	4F4C, 4M1M, 4M2S, 4M2T, 4Q9H, 4Q9I, 4Q9J, 4Q9K, 4Q9L, 4XWK, 5KPD, 5KPI, 5KPJ, 5KO2, 5KOY, 6C0V	Multidrug export
		*Cm*ABCB1	3WME, 3WMF, 3WMG, 6A6M, 6A6N	Multidrug export
		*Sc*Atm1 (ABCB7)	4MYC, 4MYH	Unknown substrate for Fe/S protein biogenesis
		TAP1/2 (ABCB2/3)	5U1D	Peptide export
		ABCB4	6S7P	Lipid export
		ABCB8	5OCH	Unknown
		ABCB10	3ZDQ, 4AYT, 4AYW, 4AYX	Unknown
		ABCB11	6LR0	Bile salt export
		ABCC subfamily		
		MRP1 (ABCC1)	5UJA, 5UJ9, 6BHU, 6UY0	Leukotriene, sphingolipid, and multidrug export
		CFTR (ABCC7)	5UAR, 5UAK, 5W81, 6D3R, 6MSM, 6O1V, 6O2P	Chloride channel
		SUR1 (ABCC8)	6BAA, 6C3O, 5YKE, 5YKF, 5YWC, 5YWD, 5YW7, 5YW8, 6JB1, 6JB3, 6PZ9,6PZA, 6PZC, 6PZI	Regulatory module of K_ATP_ channel
		ABCD subfamily		
		ABCD4	6JBJ	Cobalamin import
Type V	6 + 6 Homodimer Heterodimer Single chain	ABCA subfamily		
		ABCA1	5XJY	Phospholipid/cholesterol export
		ABCG subfamily		
		ABCG5/8	5DO7	Sterol export
		ABCG2	5NJG, 5NJ3, 6ETI, 6FEQ, 6FFC, 6HIJ, 6HCO, 6HBU, 6HZM, 6VXF, 6VXH, 6VXI, 6VXJ	Multidrug export

a Excluding ABC proteins of the ABCH and ABCI subfamilies, which most likely can be classified as type V and type III systems, respectively.

b Only PDB codes of structures with an overall resolution equal to or better than 4.5 Å were included.

## Abbreviations

ABC: ATP-binding cassette
cryo-EM: cryogenic electron microscopy
NBD: nucleotide-binding domain
TMD: transmembrane domain
