# Supplementary figures and images for: Structural and functional diversity calls for a new classification of ABC transporters
Source: FEBS Lett. Author manuscript; Available in PMC 2021 Aug 25. (PMC8386196; doi:10.1002/1873-3468.13935)

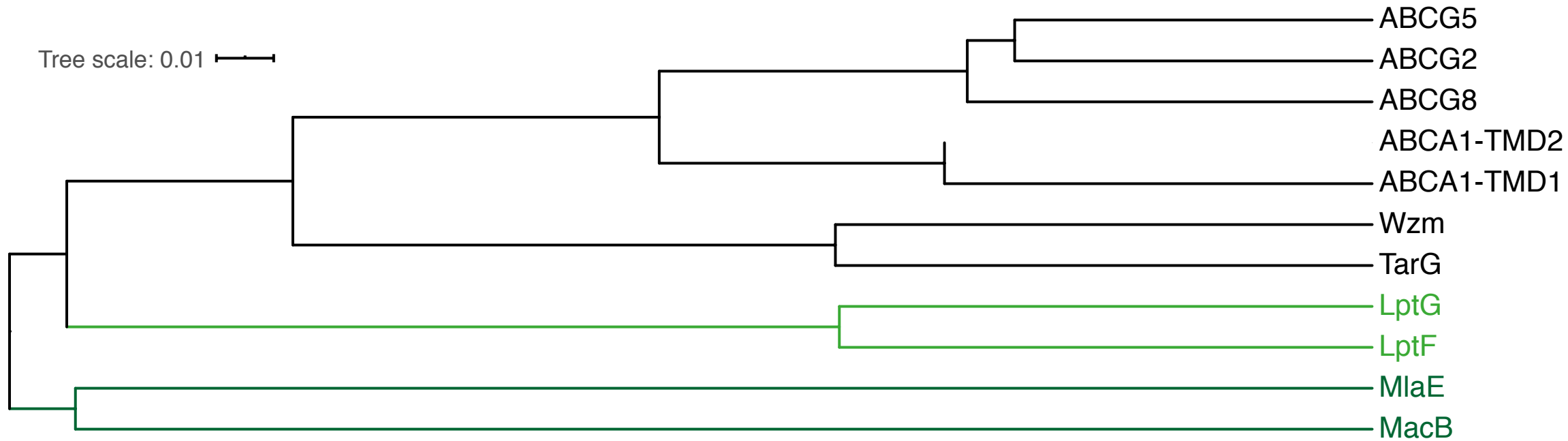

Supplement: Fig. S1. Phylogenetic tree based on TM-scores of structural TMD alignments. [file NIHMS1731057-supplement-Fig__S1__Phylogenetic_tree_based_on_TM-scores_of_structural_TMD_alignments_.pdf]
