# Supplementary material for: Structural and functional diversity calls for a new classification of ABC transporters
Source: FEBS Lett. Author manuscript; Available in PMC 2021 Aug 25. (PMC8386196; doi:10.1002/1873-3468.13935)
Supplement: Supplementary Material — Table S1. TM-scores based on pairwise structural alignment of representatives of the different TMD types. Table S2. TM-scores based on pairwise structural alignment of type I TMDs. Table S3. TM-scores based on pairwise structural alignment of type II TMDs. Table S4. TM-scores based on pairwise structural alignment of type IV TMDs in inward-facing conformations. Table S5. TM-scores based on pairwise structural alignment of type IV TMDs in (semi-) occluded/outward-facing conformations. Table S6. TM-scores based on pairwise structural alignment of type V, VI, and VII TMDsa. [file NIHMS1731057-supplement-Supplementary_Material.docx]

|  | **ArtQ (type I)** | **MolB**  **(type II)** | **CbiQ**  **(type III)** | ***Na*Atm1**  **(type IV)** | **Wzm**  **(type V)** | **LptF**  **(type VI)** | **LptG**  **(type VI)** | **MacB**  **(type VII)** |
| --- | --- | --- | --- | --- | --- | --- | --- | --- |
| **ArtQ** | 1.000 | 0.377 | 0.336 | 0.378 | 0.364 | 0.350 | 0.420 | 0.352 |
| **MolB** | 0.377 | 1.000 | 0.291 | 0.372 | 0.323 | 0.355 | 0.404 | 0.337 |
| **CbiQ** | 0.336 | 0.291 | 1.000 | 0.308 | 0.275 | 0.262 | 0.262 | 0.291 |
| ***Na*Atm1** | 0.378 | 0.372 | 0.308 | 1.000 | 0.390 | 0.354 | 0.363 | 0.534 |
| **Wzm** | 0.364 | 0.323 | 0.275 | 0.390 | 1.000 | 0.523 | 0.536 | 0.461 |
| **LptF** | 0.350 | 0.355 | 0.262 | 0.354 | 0.523 | 1.000 | 0.814 | 0.435 |
| **LptG** | 0.420 | 0.404 | 0.262 | 0.363 | 0.536 | 0.814 | 1.000 | 0.440 |
| **MacB** | 0.352 | 0.337 | 0.291 | 0.534 | 0.461 | 0.435 | 0.440 | 1.000 |

**Supplementary Table S1. TM-scores based on pairwise structural alignment of representatives of the different TMD types.**

ArtQ: 4ymu | MolB: 2nq2 | CbiQ: 5x3x | *Na*Atm1: 4mrs | Wzm: 6oih | LptF/G: 5x5y | MacB: 5ws4

**Supplementary Table S2. TM-scores based on pairwise structural alignment of type I TMDs.**

|  | **AlgM1** | **AlgM2** | **ArtQ** | **MalF** | **MalG** | **MetI** | **ModB** |
| --- | --- | --- | --- | --- | --- | --- | --- |
| **AlgM1** | 1.000 | 0.708 | 0.742 | 0.838 | 0.723 | 0.794 | 0.786 |
| **AlgM2** | 0.708 | 1.000 | 0.648 | 0.715 | 0.801 | 0.696 | 0.748 |
| **ArtQ** | 0.742 | 0.648 | 1.000 | 0.741 | 0.681 | 0.804 | 0.696 |
| **MalF** | 0.838 | 0.715 | 0.741 | 1.000 | 0.755 | 0.785 | 0.825 |
| **MalG** | 0.723 | 0.801 | 0.681 | 0.755 | 1.000 | 0.774 | 0.809 |
| **MetI** | 0.794 | 0.696 | 0.804 | 0.785 | 0.774 | 1.000 | 0.779 |
| **ModB** | 0.786 | 0.748 | 0.696 | 0.825 | 0.809 | 0.779 | 1.000 |

AlgM1/M2: 4tqu | ArtQ: 4ymu | MalF/G: 4jbw | MetI: 3tui | ModB: 3d31

**Supplementary Table S3. TM-scores based on pairwise structural alignment of type II TMDs.**

|  | **BhuU** | **BtuC** | **HmuU** | **MolB** |
| --- | --- | --- | --- | --- |
| **BhuU** | 1.000 | 0.853 | 0.864 | 0.939 |
| **BtuC** | 0.853 | 1.000 | 0.902 | 0.845 |
| **HmuU** | 0.864 | 0.902 | 1.000 | 0.855 |
| **MolB** | 0.939 | 0.845 | 0.855 | 1.000 |

BhuU: 5b57 | BtuC: 1l7v | HmuU: 4g1u | MolB: 2nq2

**Supplementary Table S4. TM-scores based on pairwise structural alignment of type IV TMDs in inward-facing conformations.**

|  | **ABCB10** | **CFTR-**  **TMD1** | **CFTR-**  **TMD2** | **IrtA** | **IrtB** | **MRP1-**  **TMD1** | **MRP1-**  **TMD2** | **MsbA** | ***Na*Atm1** | **PCAT1** | **PglK** | **Pgp-**  **TMD1** | **Pgp-**  **TMD2** | **SUR1-**  **TMD1** | **SUR1-**  **TMD2** | **TAP1** | **TAP2** | **TM287** | **TM288** | **TmrA** | **TmrB** | **YbtP** | **YbtQ** |
| --- | --- | --- | --- | --- | --- | --- | --- | --- | --- | --- | --- | --- | --- | --- | --- | --- | --- | --- | --- | --- | --- | --- | --- |
| **ABCB10** | 1.000 | 0.764 | 0.643 | 0.799 | 0.731 | 0.762 | 0.696 | 0.793 | 0.701 | 0.777 | 0.846 | 0.858 | 0.833 | 0.784 | 0.712 | 0.858 | 0.865 | 0.850 | 0.872 | 0.898 | 0.876 | 0.796 | 0.737 |
| **CFTR_TMD1** | 0.764 | 1.000 | 0.684 | 0.672 | 0.660 | 0.904 | 0.725 | 0.789 | 0.781 | 0.834 | 0.801 | 0.809 | 0.771 | 0.896 | 0.744 | 0.734 | 0.768 | 0.842 | 0.834 | 0.794 | 0.765 | 0.721 | 0.709 |
| **CFTR_TMD2** | 0.643 | 0.684 | 1.000 | 0.619 | 0.678 | 0.720 | 0.832 | 0.694 | 0.747 | 0.719 | 0.700 | 0.672 | 0.666 | 0.700 | 0.853 | 0.624 | 0.703 | 0.741 | 0.690 | 0.685 | 0.705 | 0.708 | 0.751 |
| **IrtA** | 0.799 | 0.672 | 0.619 | 1.000 | 0.640 | 0.678 | 0.678 | 0.701 | 0.618 | 0.661 | 0.740 | 0.736 | 0.701 | 0.693 | 0.692 | 0.706 | 0.791 | 0.757 | 0.772 | 0.778 | 0.792 | 0.822 | 0.684 |
| **IrtB** | 0.731 | 0.660 | 0.678 | 0.640 | 1.000 | 0.643 | 0.628 | 0.619 | 0.631 | 0.618 | 0.677 | 0.718 | 0.751 | 0.676 | 0.678 | 0.707 | 0.701 | 0.711 | 0.704 | 0.702 | 0.719 | 0.701 | 0.811 |
| **MRP1_TMD1** | 0.762 | 0.904 | 0.720 | 0.678 | 0.643 | 1.000 | 0.790 | 0.821 | 0.802 | 0.865 | 0.820 | 0.763 | 0.741 | 0.920 | 0.783 | 0.707 | 0.794 | 0.870 | 0.830 | 0.801 | 0.790 | 0.759 | 0.728 |
| **MRP1_TMD2** | 0.696 | 0.725 | 0.832 | 0.678 | 0.628 | 0.790 | 1.000 | 0.783 | 0.770 | 0.773 | 0.712 | 0.701 | 0.667 | 0.744 | 0.940 | 0.658 | 0.763 | 0.794 | 0.737 | 0.741 | 0.767 | 0.763 | 0.729 |
| **MsbA** | 0.793 | 0.789 | 0.694 | 0.701 | 0.619 | 0.821 | 0.783 | 1.000 | 0.794 | 0.848 | 0.790 | 0.764 | 0.731 | 0.809 | 0.770 | 0.749 | 0.820 | 0.850 | 0.822 | 0.844 | 0.819 | 0.776 | 0.704 |
| ***Na*Atm1** | 0.701 | 0.781 | 0.747 | 0.618 | 0.631 | 0.802 | 0.770 | 0.794 | 1.000 | 0.839 | 0.750 | 0.712 | 0.707 | 0.796 | 0.785 | 0.682 | 0.734 | 0.800 | 0.762 | 0.746 | 0.736 | 0.713 | 0.715 |
| **PCAT1** | 0.777 | 0.834 | 0.719 | 0.661 | 0.618 | 0.865 | 0.773 | 0.848 | 0.839 | 1.000 | 0.822 | 0.776 | 0.764 | 0.859 | 0.784 | 0.738 | 0.806 | 0.872 | 0.849 | 0.794 | 0.793 | 0.750 | 0.716 |
| **PglK** | 0.846 | 0.801 | 0.700 | 0.740 | 0.677 | 0.820 | 0.712 | 0.790 | 0.750 | 0.822 | 1.000 | 0.801 | 0.758 | 0.842 | 0.735 | 0.769 | 0.825 | 0.857 | 0.882 | 0.855 | 0.847 | 0.776 | 0.721 |
| **Pgp_TMD1** | 0.858 | 0.809 | 0.672 | 0.736 | 0.718 | 0.763 | 0.701 | 0.764 | 0.712 | 0.776 | 0.801 | 1.000 | 0.885 | 0.799 | 0.728 | 0.872 | 0.810 | 0.859 | 0.854 | 0.809 | 0.782 | 0.740 | 0.708 |
| **Pgp_TMD2** | 0.833 | 0.771 | 0.666 | 0.701 | 0.751 | 0.741 | 0.667 | 0.731 | 0.707 | 0.764 | 0.758 | 0.885 | 1.000 | 0.777 | 0.699 | 0.828 | 0.771 | 0.812 | 0.800 | 0.756 | 0.748 | 0.729 | 0.730 |
| **SUR1_TMD1** | 0.784 | 0.896 | 0.700 | 0.693 | 0.676 | 0.920 | 0.744 | 0.809 | 0.796 | 0.859 | 0.842 | 0.799 | 0.777 | 1.000 | 0.762 | 0.731 | 0.798 | 0.880 | 0.863 | 0.823 | 0.790 | 0.756 | 0.747 |
| **SUR1_TMD2** | 0.712 | 0.744 | 0.853 | 0.692 | 0.678 | 0.783 | 0.940 | 0.770 | 0.785 | 0.784 | 0.735 | 0.728 | 0.699 | 0.762 | 1.000 | 0.684 | 0.788 | 0.832 | 0.762 | 0.754 | 0.780 | 0.760 | 0.757 |
| **TAP1** | 0.858 | 0.734 | 0.624 | 0.706 | 0.707 | 0.707 | 0.658 | 0.749 | 0.682 | 0.738 | 0.769 | 0.872 | 0.828 | 0.731 | 0.684 | 1.000 | 0.811 | 0.805 | 0.814 | 0.782 | 0.775 | 0.714 | 0.678 |
| **TAP2** | 0.865 | 0.768 | 0.703 | 0.791 | 0.701 | 0.794 | 0.763 | 0.820 | 0.734 | 0.806 | 0.825 | 0.810 | 0.771 | 0.798 | 0.788 | 0.811 | 1.000 | 0.876 | 0.854 | 0.874 | 0.910 | 0.814 | 0.744 |
| **TM287** | 0.850 | 0.842 | 0.741 | 0.757 | 0.711 | 0.870 | 0.794 | 0.850 | 0.800 | 0.872 | 0.857 | 0.859 | 0.812 | 0.880 | 0.832 | 0.805 | 0.876 | 1.000 | 0.910 | 0.877 | 0.871 | 0.806 | 0.773 |
| **TM288** | 0.872 | 0.834 | 0.690 | 0.772 | 0.704 | 0.830 | 0.737 | 0.822 | 0.762 | 0.849 | 0.882 | 0.854 | 0.800 | 0.863 | 0.762 | 0.814 | 0.854 | 0.910 | 1.000 | 0.896 | 0.849 | 0.792 | 0.755 |
| **TmrA** | 0.898 | 0.794 | 0.685 | 0.778 | 0.702 | 0.801 | 0.741 | 0.844 | 0.746 | 0.794 | 0.855 | 0.809 | 0.756 | 0.823 | 0.754 | 0.782 | 0.874 | 0.877 | 0.896 | 1.000 | 0.928 | 0.833 | 0.764 |
| **TmrB** | 0.876 | 0.765 | 0.705 | 0.792 | 0.719 | 0.790 | 0.767 | 0.819 | 0.736 | 0.793 | 0.847 | 0.782 | 0.748 | 0.790 | 0.780 | 0.775 | 0.910 | 0.871 | 0.849 | 0.928 | 1.000 | 0.864 | 0.760 |
| **YbtP** | 0.796 | 0.721 | 0.708 | 0.822 | 0.701 | 0.759 | 0.763 | 0.776 | 0.713 | 0.750 | 0.776 | 0.740 | 0.729 | 0.756 | 0.760 | 0.714 | 0.814 | 0.806 | 0.792 | 0.833 | 0.864 | 1.000 | 0.788 |
| **YbtQ** | 0.737 | 0.709 | 0.751 | 0.684 | 0.811 | 0.728 | 0.729 | 0.704 | 0.715 | 0.716 | 0.721 | 0.708 | 0.730 | 0.747 | 0.757 | 0.678 | 0.744 | 0.773 | 0.755 | 0.764 | 0.760 | 0.788 | 1.000 |

ABCB10: 4ayw | CFTR: 5uak | IrtAB: 6tej | MRP1: 5uja | MsbA: 6bpl | *Na*Atm1: 4mrs | PCAT1: 6v9z | PglK: 5nbd | Pgp: 5ko2 | SUR1: 6jb1 | TAP1/2: 5u1d | TM287/288: 4q4h | TmrAB: 5mkk | YbtPQ: 6p6j

**Supplementary Table S5. TM-scores based on pairwise structural alignment of type IV TMDs in (semi-)occluded / outward-facing conformations.**

|  | **ABCB4-**  **TMD1** | **ABCB4-**  **TMD2** | **ABCB8** | **ABCD4** | **CFTR-**  **TMD1** | **CFTR-**  **TMD2** | **MRP1-**  **TMD1** | **MRP1-**  **TMD2** | **McjD** | **MsbA** | ***Na*Atm1** | **PglK** | **Pgp-**  **TMD1** | **Pgp-**  **TMD2** | **PrtD** | **Rv1819c** | **SUR1-**  **TMD1** | **SUR1-**  **TMD2** | **Sav1866** | **TM287** | **TM288** | **TmrA** | **TmrB** |
| --- | --- | --- | --- | --- | --- | --- | --- | --- | --- | --- | --- | --- | --- | --- | --- | --- | --- | --- | --- | --- | --- | --- | --- |
| **ABCB4-TMD1** | 1.000 | 0.931 | 0.747 | 0.740 | 0.807 | 0.769 | 0.843 | 0.862 | 0.821 | 0.778 | 0.771 | 0.785 | 0.964 | 0.909 | 0.807 | 0.771 | 0.830 | 0.793 | 0.812 | 0.821 | 0.838 | 0.801 | 0.850 |
| **ABCB4-TMD2** | 0.931 | 1.000 | 0.766 | 0.753 | 0.816 | 0.758 | 0.871 | 0.878 | 0.816 | 0.784 | 0.808 | 0.809 | 0.925 | 0.970 | 0.817 | 0.779 | 0.838 | 0.803 | 0.821 | 0.855 | 0.857 | 0.819 | 0.883 |
| **ABCB8** | 0.747 | 0.766 | 1.000 | 0.802 | 0.667 | 0.719 | 0.724 | 0.759 | 0.675 | 0.822 | 0.660 | 0.679 | 0.746 | 0.759 | 0.646 | 0.675 | 0.662 | 0.704 | 0.829 | 0.795 | 0.795 | 0.841 | 0.849 |
| **ABCD4** | 0.740 | 0.753 | 0.802 | 1.000 | 0.691 | 0.686 | 0.772 | 0.752 | 0.723 | 0.827 | 0.766 | 0.755 | 0.738 | 0.747 | 0.695 | 0.735 | 0.682 | 0.692 | 0.844 | 0.844 | 0.842 | 0.833 | 0.859 |
| **CFTR-TMD1** | 0.807 | 0.816 | 0.667 | 0.691 | 1.000 | 0.703 | 0.856 | 0.787 | 0.825 | 0.678 | 0.761 | 0.778 | 0.807 | 0.801 | 0.810 | 0.772 | 0.893 | 0.752 | 0.738 | 0.771 | 0.771 | 0.736 | 0.757 |
| **CFTR-TMD2** | 0.769 | 0.758 | 0.719 | 0.686 | 0.703 | 1.000 | 0.712 | 0.821 | 0.717 | 0.711 | 0.689 | 0.697 | 0.766 | 0.751 | 0.712 | 0.703 | 0.724 | 0.749 | 0.721 | 0.720 | 0.744 | 0.731 | 0.745 |
| **MRP1-TMD1** | 0.843 | 0.871 | 0.724 | 0.772 | 0.856 | 0.712 | 1.000 | 0.849 | 0.814 | 0.774 | 0.773 | 0.817 | 0.855 | 0.861 | 0.800 | 0.780 | 0.851 | 0.759 | 0.838 | 0.863 | 0.869 | 0.822 | 0.868 |
| **MRP1-TMD2** | 0.862 | 0.878 | 0.759 | 0.752 | 0.787 | 0.821 | 0.849 | 1.000 | 0.795 | 0.777 | 0.771 | 0.816 | 0.866 | 0.877 | 0.789 | 0.798 | 0.802 | 0.830 | 0.818 | 0.834 | 0.860 | 0.818 | 0.850 |
| **McjD** | 0.821 | 0.816 | 0.675 | 0.723 | 0.825 | 0.717 | 0.814 | 0.795 | 1.000 | 0.699 | 0.847 | 0.791 | 0.798 | 0.787 | 0.798 | 0.800 | 0.843 | 0.752 | 0.772 | 0.793 | 0.762 | 0.736 | 0.768 |
| **MsbA** | 0.778 | 0.784 | 0.822 | 0.827 | 0.678 | 0.711 | 0.774 | 0.777 | 0.699 | 1.000 | 0.718 | 0.725 | 0.774 | 0.774 | 0.668 | 0.679 | 0.684 | 0.690 | 0.893 | 0.876 | 0.865 | 0.919 | 0.881 |
| ***Na*Atm1** | 0.771 | 0.808 | 0.660 | 0.766 | 0.761 | 0.689 | 0.773 | 0.771 | 0.847 | 0.718 | 1.000 | 0.734 | 0.754 | 0.790 | 0.746 | 0.813 | 0.741 | 0.721 | 0.780 | 0.777 | 0.781 | 0.755 | 0.782 |
| **PglK** | 0.785 | 0.809 | 0.679 | 0.755 | 0.778 | 0.697 | 0.817 | 0.816 | 0.791 | 0.725 | 0.734 | 1.000 | 0.781 | 0.808 | 0.772 | 0.739 | 0.798 | 0.757 | 0.766 | 0.782 | 0.797 | 0.771 | 0.778 |
| **Pgp-TMD1** | 0.964 | 0.925 | 0.746 | 0.738 | 0.807 | 0.766 | 0.855 | 0.866 | 0.798 | 0.774 | 0.754 | 0.781 | 1.000 | 0.914 | 0.794 | 0.778 | 0.826 | 0.778 | 0.810 | 0.827 | 0.842 | 0.801 | 0.848 |
| **Pgp-TMD2** | 0.909 | 0.970 | 0.759 | 0.747 | 0.801 | 0.751 | 0.861 | 0.877 | 0.787 | 0.774 | 0.790 | 0.808 | 0.914 | 1.000 | 0.801 | 0.779 | 0.811 | 0.799 | 0.814 | 0.846 | 0.849 | 0.814 | 0.869 |
| **PrtD** | 0.807 | 0.817 | 0.646 | 0.695 | 0.810 | 0.712 | 0.800 | 0.789 | 0.798 | 0.668 | 0.746 | 0.772 | 0.794 | 0.801 | 1.000 | 0.790 | 0.855 | 0.773 | 0.724 | 0.736 | 0.756 | 0.725 | 0.730 |
| **Rv1819c** | 0.771 | 0.779 | 0.675 | 0.735 | 0.772 | 0.703 | 0.780 | 0.798 | 0.800 | 0.679 | 0.813 | 0.739 | 0.778 | 0.779 | 0.790 | 1.000 | 0.767 | 0.753 | 0.744 | 0.770 | 0.770 | 0.711 | 0.750 |
| **SUR1-TMD1** | 0.830 | 0.838 | 0.662 | 0.682 | 0.893 | 0.724 | 0.851 | 0.802 | 0.843 | 0.684 | 0.741 | 0.798 | 0.826 | 0.811 | 0.855 | 0.767 | 1.000 | 0.788 | 0.737 | 0.759 | 0.768 | 0.744 | 0.762 |
| **SUR1-TMD2** | 0.793 | 0.803 | 0.704 | 0.692 | 0.752 | 0.749 | 0.759 | 0.830 | 0.752 | 0.690 | 0.721 | 0.757 | 0.778 | 0.799 | 0.773 | 0.753 | 0.788 | 1.000 | 0.710 | 0.728 | 0.737 | 0.721 | 0.762 |
| **Sav1866** | 0.812 | 0.821 | 0.829 | 0.844 | 0.738 | 0.721 | 0.838 | 0.818 | 0.772 | 0.893 | 0.780 | 0.766 | 0.810 | 0.814 | 0.724 | 0.744 | 0.737 | 0.710 | 1.000 | 0.935 | 0.913 | 0.930 | 0.925 |
| **TM287** | 0.821 | 0.855 | 0.795 | 0.844 | 0.771 | 0.720 | 0.863 | 0.834 | 0.793 | 0.876 | 0.777 | 0.782 | 0.827 | 0.846 | 0.736 | 0.770 | 0.759 | 0.728 | 0.935 | 1.000 | 0.927 | 0.924 | 0.941 |
| **TM288** | 0.838 | 0.857 | 0.795 | 0.842 | 0.771 | 0.744 | 0.869 | 0.860 | 0.762 | 0.865 | 0.781 | 0.797 | 0.842 | 0.849 | 0.756 | 0.770 | 0.768 | 0.737 | 0.913 | 0.927 | 1.000 | 0.913 | 0.931 |
| **TmrA** | 0.801 | 0.819 | 0.841 | 0.833 | 0.736 | 0.731 | 0.822 | 0.818 | 0.736 | 0.919 | 0.755 | 0.771 | 0.801 | 0.814 | 0.725 | 0.711 | 0.744 | 0.721 | 0.930 | 0.924 | 0.913 | 1.000 | 0.927 |
| **TmrB** | 0.850 | 0.883 | 0.849 | 0.859 | 0.757 | 0.745 | 0.868 | 0.850 | 0.768 | 0.881 | 0.782 | 0.778 | 0.848 | 0.869 | 0.730 | 0.750 | 0.762 | 0.762 | 0.925 | 0.941 | 0.931 | 0.927 | 1.000 |

ABCB4: 6s7p | ABCB8: 5och | ABCD4: 6jbj | CFTR: 6msm | MRP1: 6bhu | McjD: 4pl0 | MsbA: 3b60 | *Na*Atm1: 6paq | PglK: 6hrc | Pgp: 6c0v | PrtD: 5l22 | Rv1819c: 6tqf | SUR1: 6c3o | Sav1866: 2hyd | TM287/288: 6qv0 | TmrAB: 6rah

**Supplementary Table S6. TM-scores based on pairwise structural alignment of type V, VI, and VII TMDs^a^.**

|  | **ABCA1-TMD1** | **ABCA1-TMD2** | **ABCG2** | **ABCG5** | **ABCG8** | **TarG** | **Wzm** | **LptF** | **LptG** | **MlaE** | **MacB** |
| --- | --- | --- | --- | --- | --- | --- | --- | --- | --- | --- | --- |
| **ABCA1-TMD1** | 1.000 | 0.851 | 0.795 | 0.745 | 0.771 | 0.615 | 0.612 | 0.587 | 0.563 | 0.573 | 0.550 |
| **ABCA1-TMD2** | 0.851 | 1.000 | 0.744 | 0.729 | 0.723 | 0.617 | 0.615 | 0.575 | 0.555 | 0.529 | 0.583 |
| **ABCG2** | 0.795 | 0.744 | 1.000 | 0.875 | 0.857 | 0.623 | 0.652 | 0.549 | 0.527 | 0.551 | 0.558 |
| **ABCG5** | 0.745 | 0.729 | 0.875 | 1.000 | 0.861 | 0.600 | 0.632 | 0.550 | 0.537 | 0.552 | 0.572 |
| **ABCG8** | 0.771 | 0.723 | 0.857 | 0.861 | 1.000 | 0.620 | 0.646 | 0.543 | 0.516 | 0.546 | 0.553 |
| **TarG** | 0.615 | 0.617 | 0.623 | 0.600 | 0.620 | 1.000 | 0.813 | 0.523 | 0.536 | 0.527 | 0.498 |
| **Wzm** | 0.612 | 0.615 | 0.652 | 0.632 | 0.646 | 0.813 | 1.000 | 0.523 | 0.536 | 0.500 | 0.453 |
| **LptF** | 0.587 | 0.575 | 0.549 | 0.550 | 0.543 | 0.523 | 0.523 | 1.000 | 0.814 | 0.520 | 0.435 |
| **LptG** | 0.563 | 0.555 | 0.527 | 0.537 | 0.516 | 0.536 | 0.536 | 0.814 | 1.000 | 0.502 | 0.438 |
| **MlaE** | 0.573 | 0.529 | 0.551 | 0.552 | 0.546 | 0.527 | 0.500 | 0.520 | 0.502 | 1.000 | 0.547 |
| **MacB** | 0.550 | 0.583 | 0.558 | 0.572 | 0.553 | 0.498 | 0.453 | 0.435 | 0.438 | 0.547 | 1.000 |

ABCA1: 5xjy | ABCG2: 6eti | ABCG5: 5do7 | ABCG8: 5do7 | LptF: 5x5y | LptG: 5x5y | MlaE: Coudray *et al.* | TarG: 6jbh | Wzm: 6oih

^a^TM-scores below 0.600 are highlighted in orange

**Supplementary Figure S1. Phylogenetic tree based on TM-scores of structural TMD alignments.** The phylogenetic tree was constructed based on a TM-score-based distance matrix and the UPGMA algorithm.

**Supplementary method details**

The TMDs of the various ABC systems (see [1] for MlaE structure) were analyzed for structural similarity by superposition of Cα atoms using mTM-align [2,3]. The structure-based phylogenetic tree was constructed using the TM-score-based distance matrix and the UPGMA algorithm in the PHYLIP package [4,5] and visualized with iTOL [6].

**Supplementary references**

[1] Coudray, N., Isom, G.L., MacRae, M.R., Saiduddin, M.N., Bhabha, G. and Ekiert, D.C. (2020). Structure of MlaFEDB lipid transporter reveals an ABC exporter fold and two bound phospholipids. bioRxiv, 2020.06.02.129247.

[2] Zhang, Y. and Skolnick, J. (2005). TM-align: a protein structure alignment algorithm based on the TM-score. Nucleic Acids Res 33, 2302-9.

[3] Dong, R., Peng, Z., Zhang, Y. and Yang, J. (2018). mTM-align: an algorithm for fast and accurate multiple protein structure alignment. Bioinformatics 34, 1719-1725.

[4] Sokal, R.R. and Michener, C.D. (1958). A statistical method for evaluating systematic relationships. University of Kansas Science Bulletin 38, 1409-1438.

[5] Felsenstein, J. (1989). PHYLIP - Phylogeny Inference Package (Version 3.2). Cladistics 5, 164-166.

[6] Letunic, I. and Bork, P. (2019). Interactive Tree Of Life (iTOL) v4: recent updates and new developments. Nucleic Acids Res 47, W256-W259.
